# The Continuing Emergence of *Candida blankii* as a Pathogenic Fungus: A New Case of Fungemia in a Patient Infected with SARS-CoV-2

**DOI:** 10.3390/jof8020166

**Published:** 2022-02-09

**Authors:** Ryan Mirchin, Jonathan M. Czeresnia, Erika P. Orner, Sudha Chaturvedi, Kerry Murphy, Joshua D. Nosanchuk

**Affiliations:** 1 Department of Internal Medicine, New York Presbyterian—Queens, Flushing, NY 11355, USA; mirchinr@gmail.com; 2 Department of Medicine, Division of Infectious Diseases, Montefiore Medical Center, Albert Einstein College of Medicine, Bronx, NY 10467, USA; jmambercze@montefiore.org (J.M.C.); kerry.murphy@einsteinmed.org (K.M.); 3 Department of Pathology, Montefiore Medical Center, Bronx, NY 10467, USA; eorner@montefiore.org; 4 Wadsworth Center Mycology Laboratory, New York State Department of Health, Albany, NY 12208, USA; sudha.chaturvedi@health.ny.gov

**Keywords:** *Candida blankii*, fungemia, COVID-19

## Abstract

*Candida blankii* is a recently recognized human pathogen, with most cases of the infection being reported in the immunocompromised. We here describe the case of a critically ill elderly woman with COVID-19 who developed a *C. blankii* bloodstream infection from a femoral central venous catheter. *Aspergillus niger* was also isolated from her respiratory secretions. The patient was started on voriconazole for empiric coverage of both *A. niger*, and at that time, unidentified yeast was found in the blood. Fevers persisted, and the patient expired six days after the yeast was first isolated. Almost one month after her death, *C. blankii* was identified as the cause of fungemia by sequencing of the internal transcribed spacer (ITS) region of the ribosomal gene and BLAST searching against two databases (performed by a reference laboratory). The isolate demonstrated high minimum inhibitory concentrations (MICs) to azoles and low MICs to amphotericin B, similar to previously described isolates. Timely identification of *C. blankii* would have prompted different empiric antifungal choices and possibly changed the final outcome. Clinicians should be aware of the pathological potential of *C. blankii*, the challenges of correctly identifying the organism, and its susceptibility patterns to common antifungals. There is an urgent need to improve assays for *C. blankii* identification, which will aid in accurate and timely pathogen identification, and appropriate therapeutic management.

## 1. Introduction

*Candida blankii* was first isolated over 50 years ago as a lethal pathogen in mink [1] and was subsequently isolated from artisanal cheeses and other dairy products [2]. Human infections were first described in 2015 in neonates and cystic fibrosis patients [3]. Though initially thought to be a disease of immunocompromised patients [3,4,5,6], a case of invasive disease in an immunocompetent individual has recently been described [7]. We present the second case of *C. blankii* bloodstream infection, in a critically ill patient with recent COVID-19.

## 2. Case

**Figure 1:**
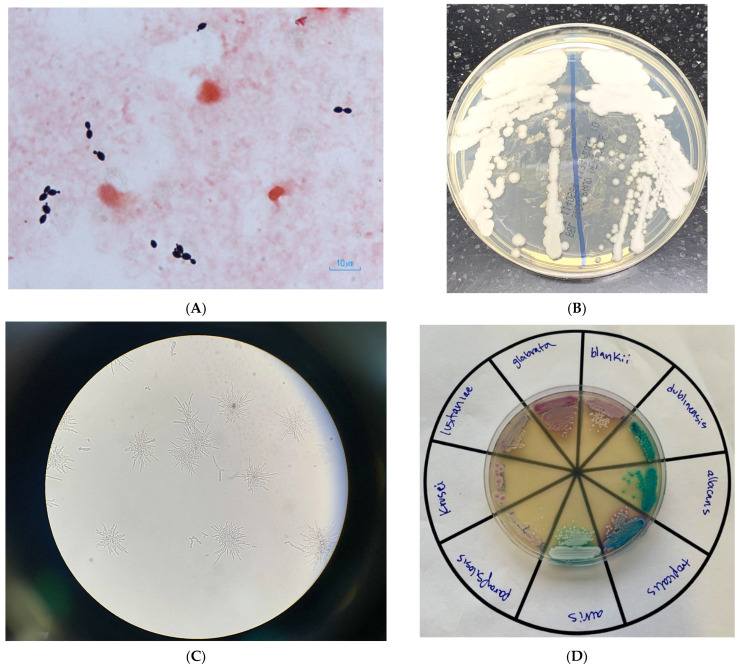
(**A**) Gram stain of blood culture containing yeast, eventually identified as *C. blankii*; (**B**) *C. blankii* growth on a Sabouraud-dextrose agar plate; (**C**) *C. blankii* pseudohyphae cultured on cornmeal agar with a polysorbate-80 plate. 100× magnification; (**D**) various *Candida* species cultured on a CHROMagr *Candida* plus agar plate that selects for and differentiates common *Candida* species.

A 76-year-old woman presented to our emergency department for low oxygen saturation. She had tested positive by polymerase chain reaction (PCR) for SARS-CoV-2 at an urgent care clinic 14 days prior, after being exposed to a household contact. Her only symptom at the time was a dry cough. Dyspnea developed in the following days, and her cough worsened. Seven days after the positive test, she developed watery diarrhea. Her medical history was notable for well-controlled hypertension, poorly controlled type 2 diabetes (glycosylated hemoglobin 12%), coronary artery disease requiring coronary artery bypass grafting several years prior, peripheral artery disease, and heart failure with preserved ejection fraction. She was a prior heavy smoker and had quit five years before admission. On arrival at our hospital, she appeared comfortable but fatigued. She was afebrile, hemodynamically stable, and oxygen saturation was 91% while breathing 100% fractional inspired oxygen through a nonrebreather mask. Physical exam was notable for crackles in both lungs and sparse rhonchi. She was admitted to the general medical floor and started on dexamethasone. Due to elevated liver enzymes and prolonged duration of disease, remdesivir was withheld. In the following days, her oxygen requirements gradually increased. On admission day 7, she was transferred to the intensive care unit and was intubated on hospital day 10. Tocilizumab was not administered, as she had developed acute renal failure requiring hemodialysis (contraindication per hospital protocol). On admission day 22, fevers of up to 39.3 °C developed. No new cavitations or nodules were noted on the portable chest X-ray. Respiratory and blood cultures were collected, and she was started on piperacillin–tazobactam and vancomycin. Tracheal aspirate cultures grew mold, which was eventually identified as *Aspergillus niger.* Peripheral blood cultures grew yeast that was identified by the BD Phoenix automated Yeast ID (YID) biochemical panel as *Trichosporon inkin.* As identification did not match fungal morphology (Figure 1; Gram-stain of blood culture smear 48 h after incubation in BD Bactec and subcultured to Sabouraud dextrose agar, cornmeal agar with polysorbate-80, and a CHROMagar *Candida* plus agar, incubated at 34–37 °C for 24–48 h), the isolate was then analyzed by matrix-assisted laser desorption/ionization–time-of-flight mass spectrometry (MALDI–TOF MS) three times, each time returning a different identification below the required probably score of 2.0 (*Malassezia furfur*, scores 1.23 and 1.34; *Empedobacter brevis,* score 1.30). Since the morphology and YID panel identification did not match, and since the isolate could not be identified by MALDI–TOF MS, the isolate was sent to the Wadsworth Center Mycology Laboratory, New York State Department of Health, for identification. The source of fungemia was believed to be a femoral central venous catheter. The patient was started on voriconazole for empiric coverage of both *A. niger* and the then-unidentified yeast. She remained febrile despite the antifungal. The femoral venous catheter was removed. She was unable to be weaned off the ventilator and underwent a tracheostomy on hospital day 26. Hemodynamic instability developed two days later, following an episode of supraventricular tachycardia (SVT), at which point family members elected to not further escalate care. She expired on hospital day 30, six days after the yeast was first isolated from her blood. Given the persistence of fevers, her mycoses were considered to have contributed to the episode of SVT, which eventually led to her final demise. Almost one month after the patient expired, *Candida blankii* was identified as the cause of fungemia by sequencing of the internal transcribed spacer (ITS) region of the ribosomal gene and BLAST searching against two databases: National Center for Biotechnology Information (https://blast.ncbi.nlm.nih.gov/Blast.cgi (accessed on 11 October 2021) and CBS-KNAW (https://wi.knaw.nl/page/Pairwise_alignment (accessed on 11 October 2021)), with 100% identity. The ITS sequence of *C. blankii* was deposited in GenBank with accession number OL697232. The antifungal susceptibility testing (AFST) was performed on *C. blankii* isolate by M27-A3 CLSI broth microdilution. The isolate had higher minimum inhibitory concentration (MIC) to fluconazole (256 mg/mL), voriconazole (8 mg/mL), posaconazole (2 mg/mL), isavuconazole (1 mg/mL), and itraconazole (1 mg/mL). Anidulafungin had high MIC (2 mg/mL), followed by micafungin (0.5 mg/mL), and caspofungin (0.25 mg/mL). The isolate was susceptible to amphotericin B with a MIC of 0.064 mg/mL.

## 3. Discussion

Characteristics of cases reported to date are outlined in Table 1. *C. blankii* seems to have pathogenic potential in three distinct age groups—as a bloodborne pathogen in neonates [4,5], as a lung colonizer with pathogenic potential in adolescents with structural lung disease [3,6], and finally, again as a cause of fungemia in adults [7]. In our patient, the femoral central venous catheter is likely to have been the source of fungemia. Dysbiosis caused by infection with SARS-CoV2 [8] and previous broad-spectrum antibiotic use may also have contributed to the development of candidemia.

As is evident in the case of our patient, who had two different fungi isolated from bodily fluids during admission for severe COVID-19, we have seen a steep increase in the incidence of cases of invasive mycosis since the onset of the pandemic [9]. Examples include the following cases:

- Aspergillosis: COVID-19-associated pulmonary aspergillosis (CAPA) occurred in over 30% of cases admitted to intensive care units (ICUs) in some publications [10], though more robust meta-analyses suggest the true incidence being closer to 6% [11];
- Mucormycosis: as of June 2021 (latest available data), over 40,000 cases of mucormycosis complicating SARS-CoV-2 infection had been reported to the Indian government [12]. Cases have been described in over 18 countries [13];
- Candidiasis: invasive *Candida* infections, including those caused by *C. auris*, have been reported [14,15];
- Others: cases of *Saccharomyces cerevisiae* [16] and *Trichosporon asahii* fungemia [17] complicating SARS-CoV-2 infection have been reported.

Data from seven isolates of *C. blankii* submitted to the United Kingdom’s National Mycology Reference Laboratory (2002–2016) showed that almost all of them had reduced susceptibility to azoles, while none of them were resistant to amphotericin B or flucytosine [18]. Similar trends can be noted in most isolates reported in Table 1, including the one that infected our patient. Though research comparing outcomes between antifungals in *C. blankii* infection is lacking, these data suggest that amphotericin B should be the antimicrobial of choice when empirically treating invasive *C. blankii*. In our case, timelier identification of *C. blankii* may have prompted the use of amphotericin B in lieu of voriconazole and could have possibly changed the final outcome.

Notably, almost half a century elapsed since the first description of *C. blankii* and its identification as a human pathogen. Though this delay is likely, in part, attributable to challenges in isolating and correctly identifying the organism, global warming may be contributing to the emergence of *C. blankii* as a human pathogen, similar to what has been proposed as a cause for the nearly simultaneous emergence of different clades of pathogenic *C. auris* in different continents [19].

Though the widespread use of corticosteroids and biologic immunomodulatory agents is believed to be contributing to the increase in the incidence of invasive fungal disease [20], severe infection with SARS-CoV-2 itself has been shown to impair immune response against *C. albicans* in vitro; however, the same was not true for *Aspergillus fumigatus* [21].

While the mechanisms that explain the high frequency of fungal infections in patients with COVID-19 have yet to be elucidated, the explosive increase in the incidence of these infections is clear. Our case now adds another organism, *C. blankii*, to the growing list of fungi implicated in complicating infection with SARS-CoV-2.

## 4. Conclusions

Ongoing vigilance, including efforts to correctly identify uncommon organisms, is paramount, as new fungal scourges are expected to continue to arise with ongoing changes in our environments and increased use of immunosuppressive medications including steroids and immunomodulators in clinical practice. Clinicians should be aware of the pathological potential of *C. blankii*, the challenges of correctly identifying the organism, and its susceptibility patterns to common antifungals. Our case illustrates how *C. blankii* infection can complicate cases of COVID-19. More research is needed to further explore the role of this fungus in human disease. The current method of accurate identification of *C. blankii* is limited to sequencing the ribosomal genes, followed by BLAST search. Such technologies are not readily available to resource-poor or low complexity clinical laboratories. There is an urgent need to improve biochemical or protein-based assays for *C. blankii* identification, which will aid in accurate and timely pathogen identification, and appropriate therapeutic management.

## Figures and Tables

**Table 1:** Reported cases of *C. blankii* infection and colonization.

Patient (Reference)	Age at Dx	Sex	Location of Case	Medical History/Comorbidities	Infection Site	Susceptibilities of Strain(s) μg/mL	Treatment Modality	Patient Outcome
1 (3)	14 y	M	Argentina	Cystic fibrosis	Respiratory colonization leading to respiratory failure	<0.13 for amphotericin B, fluconazole, voriconazole, itraconazole, posaconazole, anidulafungin and caspofungin	Itraconazole 200 mg daily → 100 mg	Recovered
2 (12)	16 y	F	Brazil	Cystic fibrosis status post bilateral lung transplantation	Fungemia	Fluconazole: 16 Voriconazole: 0.5 Amphotericin B: 0.25–0.5 Anidulafungin: 0.25–1 Micafungin: 0.5–1	Micafungin 100 mg daily × 14 days	Recovered
3 (4)	27 w **	M	India	Preterm birth, necrotizing enterocolitis	Fungemia	Fluconazole: 12–16 Voriconazole: 0.19–0.38 Itraconazole: 0.75 Posaconazole: 0.5–0.75 Amphotericin B: 0.19–0.38 Caspofungin: 0.25–0.5 Micafungin: 0.125 Anidulafungin: 0.19	Amphoteicin B and caspofungin	Deceased
4 (5)	2–3 d **	M	India	VLBW, IUGR, sepsis, CVC, severe asphyxiation, mech vent, venous thrombosis	Fungemia	*Mean MICs of isolates* Fluconazole: 8 Isavuconazole: 0.07 Posaconazole: 0.13 Itraconazole: 0.18 Voriconazole: 0.25 Anidulafungin: 2 Micafungin: 0.06	Fluconazole × 10 days *	Deceased
5 (5)	2–3 d **	M	India	LBW, IUGR, sepsis	Fungemia		Fluconazole × 14 days *	Recovered
6 (5)	2–3 d **	F	India	Preterm, LBW, sepsis	Fungemia		Fluconazole × 14 days *	Recovered
7 (5)	2–3 d **	F	India	Preterm, LBW, IUGR	Fungemia		Fluconazole × 12 days *	Recovered
8 (5)	2–3 d **	F	India	VLBW, sepsis, severe asphyxiation, CVC, mech vent, hypoglycemia	Fungemia		Fluconazole × 6 days *	Deceased
9 (5)	2–3 d **	M	India	Early preterm, ELBW, severe asphyxiation, CVC, sepsis, mech vent	Fungemia		Fluconazole × 10 days *	Deceased
10 (5)	2–3 d **	M	India	Early preterm, VLBW, sepsis, hypoglycemia, severe asphyxiation, CVC, mech vent	Fungemia		Fluconazole × 10 days *	Recovered
11 (5)	2–3 d **	M	India	Severe asphyxiation, hypoglycemia, mech vent, CVC	Fungemia		Fluconazole × 5 days *	Deceased
12 (5)	2–3 d **	M	India	Early preterm, ELBW, severe asphyxia, sepsis, CVC, mech vent	Fungemia		Fluconazole × 21 days *	Recovered
13 (7)	63 y	M	USA	HTN, HLD, DM2, sepsis, perinephric abscess, endocarditis with new embolic strokes	Fungemia	Fluconazole: 16 Itraconazole: 0.5 Posaconazole: 1 Voriconazole: 0.250 Amphotericin B: 0.5 Anidulafungin: 0.250 Caspofungin: 1 Micafungin: 0.120 5-Flucytosine: <0.06	Amphotericin B and micafungin × 12w, then voriconazole suppression × 9m	Recovered
14 (our case)	76 y	F	USA	HTN, DM2, peripheral artery disease, HFpEF	Fungemia	Fluconazole: 256 Itraconazole: 1 Voriconazole: 8 Posaconazole: 2 Caspofungin: 0.25 Micafungin: 0.5 Amphotericin B: 0.064	Voriconazole	Deceased

* All neonates received fluconazole 12 mg/kg body weight (loading dose), followed by 6 mg/kg body weight first. ** date of fungemia onset for each neonate not specified, all were at 2–3d of life. LBW, low birth weight (<2500 g); VLBW, very low birth weight (<1500 g); ELBW, extremely low birth weight (<1000 g); IUGR, intrauterine growth restriction; CVC, cardiovascular collapse; Mech Vent, mechanical ventilation; HTN, hypertension; HLD, hyperlipidemia; DM2, type 2 diabetes mellitus; HfpEF, heart failure with preserved ejection fraction.
